# Upregulated miR-18a-5p in Colony Forming Unit-Hill’s in Subclinical Cardiovascular Disease and Metformin Therapy; MERIT Study †

**DOI:** 10.3390/biomedicines10092136

**Published:** 2022-08-31

**Authors:** Jason Phowira, Fahad W. Ahmed, Sherin Bakhashab, Jolanta U. Weaver

**Affiliations:** 1 Translational and Clinical Research Institute, Newcastle University, Newcastle upon Tyne NE2 4HH, UK; 2 Faculty of Medicine, Universitas Indonesia, Jakarta 10430, Indonesia; 3 Department of Diabetes, Queen Elizabeth Hospital, Gateshead, Newcastle upon Tyne NE9 6SH, UK; 4 Department of Medical Oncology, King Faisal Specialist Hospital and Research Centre, Madinah 42522, Saudi Arabia; 5 Biochemistry Department, King Abdulaziz University, P.O. Box 80218, Jeddah 21589, Saudi Arabia; 6 Vascular Biology and Medicine Theme, Newcastle University, Newcastle upon Tyne NE1 7RU, UK

**Keywords:** miR-18a-5p, CFU-Hill’s colonies, subclinical CVD, metformin

## Abstract

Colony forming unit-Hill’s (CFU-Hill’s) colonies are hematopoietic-derived cells that participate in neovasculogenesis and serve as a biomarker for vascular health. In animals, overexpression of miR-18a-5p was shown to be pro-atherogenic. We had shown that well-controlled type 1 diabetes mellitus (T1DM) is characterized by an inflammatory state, endothelial dysfunction, and reduced number of CFU-Hill’s, a model of subclinical cardiovascular disease (CVD). MERIT study explored the role of miR-18a-5p expression in CFU-Hill’s colonies in T1DM, and the cardioprotective effect of metformin in subclinical CVD. In T1DM, miR-18a-5p was significantly upregulated whereas metformin reduced it to HC levels. MiR-18a-5p was inversely correlated with CFU-Hill’s colonies, CD34+, CD34+CD133+ cells, and positively with IL-10, C-reactive protein, vascular endothelial growth factor-D (VEGF-D), and thrombomodulin. The receiver operating characteristic curve demonstrated, miR-18a-5p as a biomarker of T1DM, and upregulated miR-18a-5p defining subclinical CVD at HbA1c of 44.5 mmol/mol (pre-diabetes). Ingenuity pathway analysis documented miR-18a-5p inhibiting mRNA expression of insulin-like growth factor-1, estrogen receptor-1, hypoxia-inducible factor-1α cellular communication network factor-2, and protein inhibitor of activated STAT 3, whilst metformin upregulated these mRNAs via transforming growth factor beta-1 and VEGF. We confirmed the pro-atherogenic effect of miR-18a-5p in subclinical CVD and identified several target genes for future CVD therapies.

## 1. Introduction

Cardiovascular disease (CVD) encompasses a group of diseases affecting the heart and blood vessels; these include coronary heart disease (CHD), cerebrovascular disease, peripheral arterial disease, rheumatic heart disease, congenital heart disease, deep vein thrombosis, and pulmonary embolism [1]. CVD remains the major cause of death globally, with an estimated 17.9 million CVD-related deaths each year [1]. As over 80% of premature CVD is preventable, identifying people at risk of CVD plays an important role in preventing and managing CVD [2]. Type 1 diabetes mellitus (T1DM) has been associated with elevated risks of CVD and premature mortality [3]. CVD appears as the major cause of morbidity and mortality among patients with longstanding T1DM, contributing to approximately 11–13-year shorter life expectancy in comparison to healthy adults [4]; moreover, even T1DM patients with controlled glycated hemoglobin (HbA1c) of 52 mmol/mol or lower were nearly three times as likely to die as a result of CVD, and the risks were substantially higher among individuals with higher levels of HbA1c [5]. 

We and others have demonstrated that T1DM can be considered as subclinical CVD, due to the presence of endothelial dysfunction, elevated levels of inflammatory markers, higher levels of circulating endothelial cells (ECs), and reduced levels of circulating endothelial progenitor cells (cEPCs), colony forming units (Hill’s) and pro-angiogenic cells (PACs) [6,7,8,9,10,11]. 

Several approaches have been utilized to identify and isolate early outgrowth EPCs, one of which involves the colony-forming ability of the plated mononuclear cells. The original method was introduced by Asahara et al. [12] and was expanded by Ito et al. [13]. In 2003, Hill et al. further modified this cluster assay method, measuring a mixture of hematopoietic cells, including lymphocytes, monocytes, and hematopoietic progenitor cells [8,14]; this assay has become known as the colony-forming unit-Hill (CFU-Hill’s) colony assay [8]. CFU-Hill’s colonies are hematopoietic-derived cells that are unable to form vascular structures in vivo; however, it was suggested that these cell populations may participate in neovasculogenesis in a paracrine manner, which is likely by secreting angiogenic cytokines that activate mature ECs and enhance vasculogenesis [15,16]; moreover, Yoder et al. reported that majority of CFU-Hill’s colonies expressed CD115, a known receptor for colony-stimulating factor 1 which plays a crucial role in vascular endothelial growth factor (VEGF) production, EPC mobilization, and angiogenesis in vivo [16]. 

Hill et al. demonstrated a significant inverse correlation between circulating CFU-Hill’s colonies concentration and the Framingham risk score for the assessment of the total burden of risk factors of CHD at 10 years [8]; moreover, subjects with elevated serum cholesterol levels, hypertension or diabetes mellitus demonstrated significantly lower levels of CFU-Hill’s colonies compared to healthy subjects [8]. Salazar-Martinez et al. studied 49 children and teenagers aged between 10 and 17, and found inverse associations between the formation of CFU-Hill’s colonies and obesity, dyslipidemia, and high blood glucose levels [17]. Furthermore, independent of cardiovascular risk factors, CFU-Hill’s colonies have been demonstrated to be about 40% lower in obese and overweight compared to normal weight individuals [18]. 

Additionally, it was also observed that subjects with diabetes, hypertension and elevated cholesterol concentrations demonstrated significantly lower numbers of CFU-Hill’s colonies [8]; these findings support the role of CFU-Hill’s colonies as a potential biomarker for early detection and management of CVD. The lack of research into CFU-Hill’s colonies is likely to be related to the paucity of material amenable to previously available laboratory techniques. Nevertheless, it offers new avenues for CVD research.

Metformin, a widely-used anti-diabetic drug, has been demonstrated to not only effectively lower blood glucose but also reduce cardiovascular-related mortality and the incidence of cardiovascular events [19]. Metformin may exert its anti-atherogenic effect by correcting some atherosclerotic risk factors (blood pressure, body weight, triglycerides and LDL cholesterol, glycemic control, insulin resistance) and protecting the arteries from fibrosis and remodeling [20]. Metformin has been demonstrated to preserve endothelial function, therefore exerting its protective effect on diabetic microangiopathies; this is supported by the direct role of metformin in mitigating the deleterious effects of diabetes on endothelial, including increased production of ROS, mitochondrial dysfunction, generation of advanced glycation end-products (AGEs), endothelial apoptosis and senescence, activation of the polyol pathway, and dysregulation of microRNAs (miRNAs) [21]. In animal models of myocardial infarction, it was demonstrated that metformin administration, given at reperfusion, reduced myocardial infarct size in nondiabetic rats [22]; moreover, we have shown the cardioprotective effect of metformin in patients with T1DM through improvement in CFU-Hill colonies, circulating EPCs, circulating ECs, and PACs whilst glycemic control was unchanged [6]. Emerging studies have suggested the utilization of miRNAs, a class of small noncoding RNA molecules, in the evaluation of health status and disease progression [23]. Currently, miRNAs have been acknowledged to play a part in the regulation of aging, apoptosis, proliferation, metabolism, cellular differentiation, and pathogenesis of diseases including CVD [24]. 

Accumulating evidence has suggested that the dysregulation of multiple miRNAs is responsible for the pathogenesis of CVD. The overexpression of miRNA-18a-5p has been identified as pro-atherogenic miRNA as it promotes proliferation and migration of vascular smooth muscle cells [25,26]; moreover, an animal model of oxygen-induced proliferative retinopathy revealed that the overexpression of miR-18a-5p in human retinal microvascular endothelial cell, was anti-angiogenic as inhibited angiogenesis via significant downregulation of fibroblast growth factor-1 and hypoxia-inducible factor-1 alpha (HIF-1α), which are both closely associated with angiogenesis [27]. The anti-angiogenic property of miR-18a-5p renders it an ideal candidate for studying the paracrine function of CFU-Hill’s colonies.

We hypothesized that miRNA-18a-5p expression in CFU-Hill’s colonies is upregulated in T1DM and is downregulated by metformin therapy. By validating the anti-angiogenic role of miRNA-18a-5p in CFU-Hill’s colonies in patients, we could therefore explore therapeutic pathways or targets to manage CVD.

## 2. Materials and Methods

### 2.1. Subjects 

Twenty-nine patients with T1DM of 22.4 ± 13.9 years duration and no overt CVD and 20 matched healthy controls (HCs) were recruited in the present study to explore the role of miR-18a-5p in subclinical CVD in T1DM. The inclusion criteria of our study were T1DM patients with HbA1c < 8.5% (69 mmol/mmol), absence of macrovascular disease or renal impairment. Metformin was administered to T1DM patients for 8 weeks with a dose titrated up to a maximum of 1 g twice a day or to the highest tolerated dose. 

The study was conducted in accordance with the Helsinki Declaration and all subjects gave written informed consent prior to their inclusion in the study. The study was approved by the NHS Health Research Authority, NRES Committee Northeast Sunderland, UK (Research Ethics Committee Reference Number 12/NE/0044).

### 2.2. Meso Scale Discovery (MSD) Assay

To measure cytokine levels, plasma samples were diluted according to the manufacturer’s instruction from the study groups and were assayed using K15050D V-PLEX Cytokine Panel 1 human kit, K15049D V-PLEX Proinflammatory Panel 1 human kit, K15135C Human Vascular Injury 1 kit, K15198D V-PLEX Vascular Injury Panel 2 human kit and K15190D V-PLEX Angiogenesis Panel 1 human kit (Meso Scale Discovery, Rockville, MD, USA) in accordance with the manufacturer’s protocol.

### 2.3. Flow Cytometric Evaluation of Circulating Endothelial Progenitor Cells

As previously described, the cEPCs, also defined as CD45^dim^CD34^+^CD133^+^ cells, were analyzed using flow cytometry on a BD FACS Canto^TM^ II system (BD Bioscience, San Jose, CA, USA) [6]. The cEPC and circulating progenitor cells were labeled with a collection of antibodies (BD Bioscience, Table 1).

### 2.4. Culture and Quantification of CFU-Hill’s Colonies

CFU-Hill’s colonies were cultured according to the method described by Hill et al., 2003 [8]. Others also used this method of culturing CFU-Hills [16,17,18,28]. Briefly, 5 × 10^6^ peripheral blood mononuclear cells (PBMNC) were plated on a fibronectin-coated 6-well culture plate. The cultured plate was washed twice with PBS to obtain all non-adherent cells. The cell suspension from the supernatant was centrifuged at 500× *g* for 5 min at room temperature (deceleration = 3). The supernatant was discarded, and the cell pellet was re-suspended in 1 mL of medium and counted. 1 × 10^6^ cells were re-plated in fresh fibronectin-coated in 3 wells of 24-well plates in the complete endothelial basal medium. The plates were incubated at 37 °C in 5% CO_2_/air, and the medium was changed every three days. On day seven, colonies showing features of multiple spindle-shaped cells originating from a central cluster of round cells were identified as CFU-Hills colonies. Three wells were washed twice with PBS to remove non-CFU-Hills cells. An important feature of these colonies is the fact that CFU-Hills disappear within 10–14 days [29]. The CFU-Hills cells were lysed in wells with lysis buffer for further analysis. The mean number of CFU-Hills per well was in control subjects 20.5 and 8.5 in patients before metformin therapy and 13.8 after metformin therapy. 

### 2.5. Plasma Sample Preparation

Platelet-free plasma was obtained through consecutive centrifugations of blood samples at 500× *g* for 15 min and 13,000× *g* for another 5 min. The plasma was tested for haemolysis to ensure the samples were not contaminated with cellular miRNA. An aliquot of 200 μL per sample was transferred to a FluidX tube and 60 μL of Lysis solution BF containing 1 μg carrier-RNA per 60 μL Lysis Solution BF and RNA spike-in template mixture was added to the sample and mixed for 1 min and incubated for 7 min at room temperature, followed by addition of 20 μL Protein Precipitation solution BF. Total RNA was extracted from the samples using miRCURY RNA isolation Kit—Biofluids, high-throughput bead-based protocol v.1 (Exiqon, Vedbaek, Denmark) in an automated 96-well format. The purified total RNA was eluted in a final volume of 50 μL. Plasma miRNAs were isolated from plasma samples using an RNA isolation protocol optimized for plasma by QIAGEN (Exiqon Services, Vedbeaek, Denmark). The integrity of extracted RNAs were measured using 2100 Bioanalyzer (Agilent, Santa Clara, CA, USA) with the value of 9.1–10 considered as high in integrity.

### 2.6. MiRNA Expression Using Real-Time Quantitative PCR

Purification of total RNA was performed using miRNeasy Micro Kit (QIAGEN, Hilden, Germany). The cell pellet was lysed in 700 µL QIAzol and 1.25 µL of each: UniSpike 2, 4, 5 and MS2 carrier RNA (Roche, Basel, Switzerland) was directly added to the QIAzol lysate. The sample was then vortexed and kept in the dark for 5 min to ensure the samples were completely lysed. An additional DNase on-column treatment (QIAGEN, Hilden, Germany) was performed according to the manufacturer’s instructions.

Approximately 10 μL RNA was reverse transcribed in 50 μL reactions using the miRCURY LNA RT Kit (QIAGEN, Hilden, Germany). cDNA was diluted 100× and assayed in 10 μL PCR reactions according to the protocol for miRCURY LNA miRNA PCR; miRNA was assayed once by qPCR on the miRNA Ready-to-Use PCR, Human panel I + II (Catalog number: 339322, QIAGEN) using miRCURY LNA SYBR Green master mix. The amplification was performed in a LightCyclerR 480 Real-Time PCR System (Roche, Basel, Switzerland) in 384 well plates. The amplification curves were analyzed using the Roche LC software 4, Basel, Switzerland, both for the determination of Cq (by the 2nd derivative method) and for melting curve analysis. All Cq data was normalized using the global mean method based on the average of the assays detected in all samples, yielding ΔCq values. Fold-change analysis was performed using 2 × |ΔΔCq| calculation, with ΔΔCq obtained from (∆Cq × T1DM) − (∆Cq × HCs).

### 2.7. Pathway Analysis: Ingenuity Pathway Analysis of miR-18a-5p

Target genes, cellular functions, and pathological pathways regulated by miR-18a-5p were predicted by using ingenuity pathway analysis (IPA) software 9.0 (Ingenuity, Redwood City, CA, USA). In comparison, the interaction sites between the transcripts and miR-18a-5p was detected by TargetScan Human release 7.1 (www.targetscan.org, accessed on 4 April 2022) and Diana-TarBase v8 (http://carolina.imis.athena-innovation.gr/diana_tools/web/index.php?r=tarbasev8%2Findex, accessed on 4 April 2022) databases.

### 2.8. Statistical Analysis

Data were presented as mean ± standard deviation, unless stated otherwise. To assess the normality of the data, Shapiro–Wilk test was performed. The unpaired *t*-test or Mann-Whitney test was used to measure the difference between the groups. The correlation between miR-18a-5p expression and other markers was assessed using linear regression tests. Receiver Operating Characteristic (ROC) curve analysis was performed to assess the sensitivity of miR-18a-5p as a biomarker for T1DM (subclinical CVD) and to establish the cut-off value for miR-18a-5p upregulation. Statistical analyses were performed using IBM^TM^ SPSS^TM^ software Version 24.0 (SPSS^TM^ Inc., Armonk, NY, USA) and the graphs were constructed using GraphPad Prism 9.0 (GraphPad software, San Diego, CA, USA). A *p*-value less than 0.05 was considered statistically significant. 

## 3. Results

### 3.1. Characteristics of Studied Subjects

The present study enrolled 29 T1DM patients and 20 age- and gender-matched HCs. The demographic and clinical characteristics of the participants were published previously [30]. The mean age of the T1DM group and HCs were 47.2 and 46.5, respectively. The T1DM group had relatively good glycemic control with a mean HbA1c of 57.3 mmol/mol (7.4%).

### 3.2. Comparison of the Levels of Cytokines, C-Reactive Protein (CRP), and Vascular Health Indices between HC and T1DM

**Figure 1:**
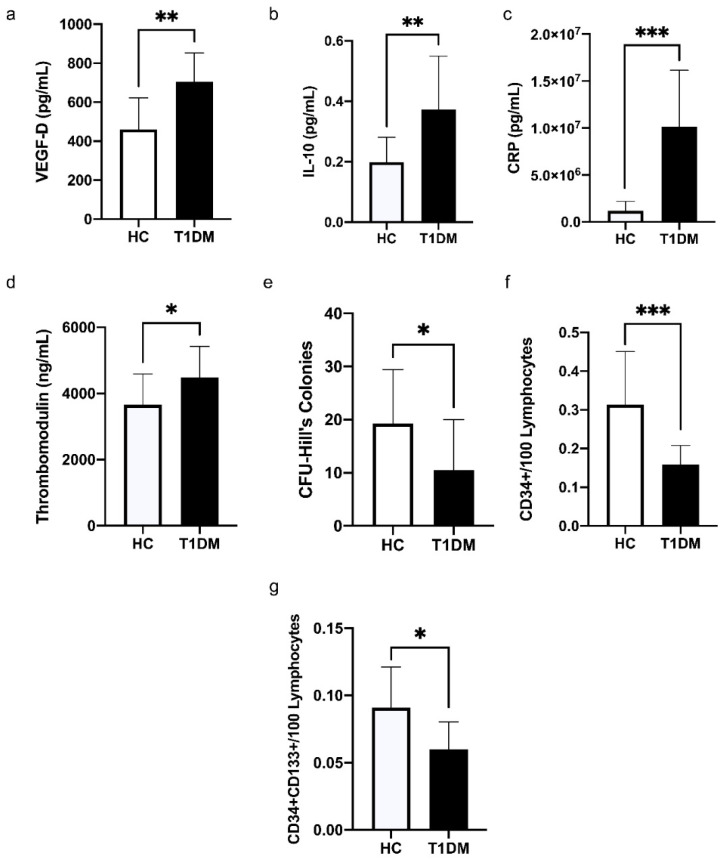
The comparison of data between HC and T1DM patients in terms of the levels of (**a**) VEGF-D in plasma, (**b**) IL-10 in plasma, (**c**) CRP in plasma, (**d**) thrombomodulin in plasma, (**e**) CFU-Hill’s colonies, (**f**) CD34^+^/100 lymphocytes, and (**g**) CD34^+^CD133^+^/100 lymphocytes. Data are presented as means ± SD and analyzed by unpaired *t*-test or Mann-Whitney U test. VEGF: vascular endothelial growth factor; IL: interleukin; CRP: C-reactive protein; CFU: colony-forming unit; CD: a cluster of differentiation. * *p* < 0.05; ** *p* < 0.01; *** *p* < 0.001.

In T1DM patients, the levels of VEGF-D (*p* = 0.002, Figure 1a) and IL-10 (*p* = 0.008, Figure 1b) were significantly higher in comparison to HCs. The levels of CRP in plasma were detected to be significantly upregulated in patients with T1DM compared to HCs (*p* < 0.001, Figure 1c).

T1DM patients presented significantly higher level of thrombomodulin (*p* = 0.046, Figure 1d), and significantly lower level of CFU-Hill’s colonies (*p* = 0.04, Figure 1e) in comparison to HCs; moreover, the levels of circulating progenitor cells, CD34^+^/100 lymphocytes (Figure 1f) and CD34^+^CD133^+^/100 lymphocytes (Figure 1g), were both significantly lower among T1DM patients (*p* = <0.001, *p* = 0.013).

We have previously published that metformin therapy resulted in significantly increased CFU-Hill’s colonies count, which was closer to the levels seen in HC [6].

All comparisons were independent experiments thus correction for multiple comparisons has not been applied. The effects of metformin on vascular indices have been published before [6]. 

### 3.3. The Expression of miR-18a-5p in Study Participants

In the T1DM patient group, the expression of miR-18a-5p in the CFU-Hill’s colonies was demonstrated to be significantly upregulated versus HC (*p* = 0.008), whilst in plasma no difference in the expression of miR-18a-5p in T1DM versus HC was detected. 

**Figure 2:**
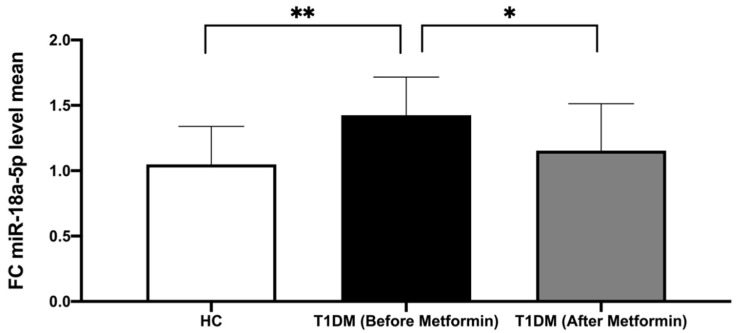
Comparison of miR-18a-5p expression in healthy controls and T1DM patients before and after metformin. Data are presented as means ± SD and the difference between groups is analyzed by *t*-test; * *p* < 0.05; ** *p* < 0.01.

Following metformin therapy, we observed a significant decrease in miR-18a-5p expression in T1DM (*p* = 0.044), normalizing its expression in comparison to HC (*p*-value non-significant, Figure 2).

**Figure 3:**
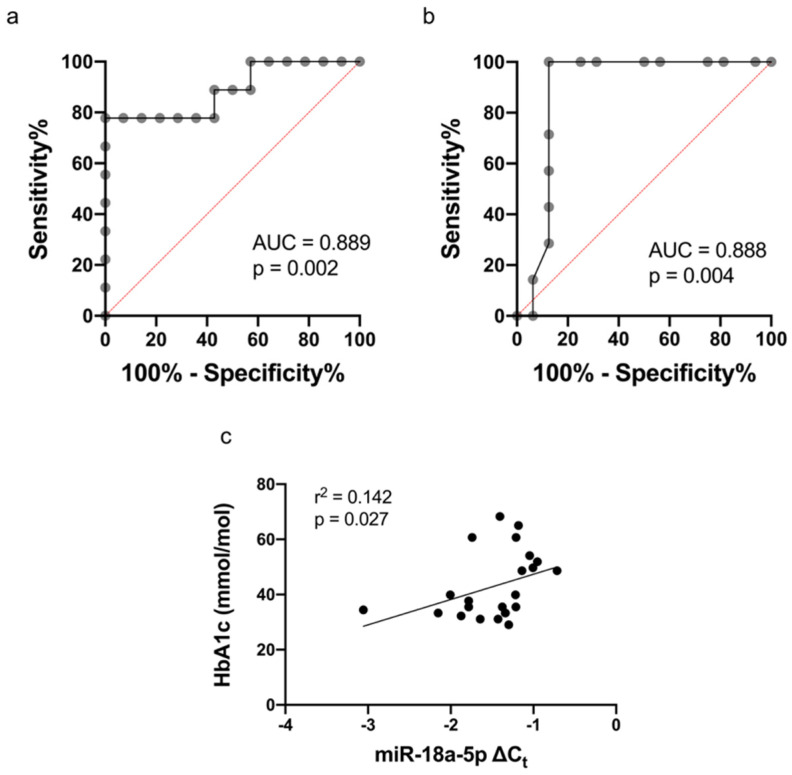
(**a**) Receiver operating characteristic (ROC) curve analysis of miR-18a-5p in discriminating T1DM patients from healthy controls; (**b**) ROC curve analysis of HbA1c to indicate upregulated miR-18a-5p expression in CFU-Hill’s colonies; (**c**) Correlation between miR-18a-5p in CFU-Hill’s colonies and HbA_1c_ as a parameter to evaluate glycemic control (r^2^ = 0.142, *p* = 0.027). ROC curve analysis was performed to determine optimal cut-off values, whilst linear regression analysis was performed to assess the correlation between miR-18a-5p and HbA_1c_. HbA1c: glycated hemoglobin.

Furthermore, ROC analysis of miR-18a-5p demonstrated area under the curve (AUC) of 0.889 (*p* = 0.002) with cut-off value of −1.211 (specificity = 100% and sensitivity = 77.78%, Figure 3a). Additional ROC analysis was performed to determine the level of HbA1c at which miR-18a-5p was upregulated with AUC of 0.888 (*p* = 0.004) and HbA1c cut-off value 44.5 mmol/mol (6.2%), specificity = 87.5% and sensitivity = 100% (Figure 3b). There was significant correlation between miR-18a-5p in CFU-Hill’s colonies and HbA_1c_ before the use of metformin (r^2^ = 0.142, *p* = 0.027) as shown in Figure 3c.

### 3.4. Correlations of miR-18a-5p with Cytokines, CRP, and Parameters of Vascular Function

**Figure 4:**
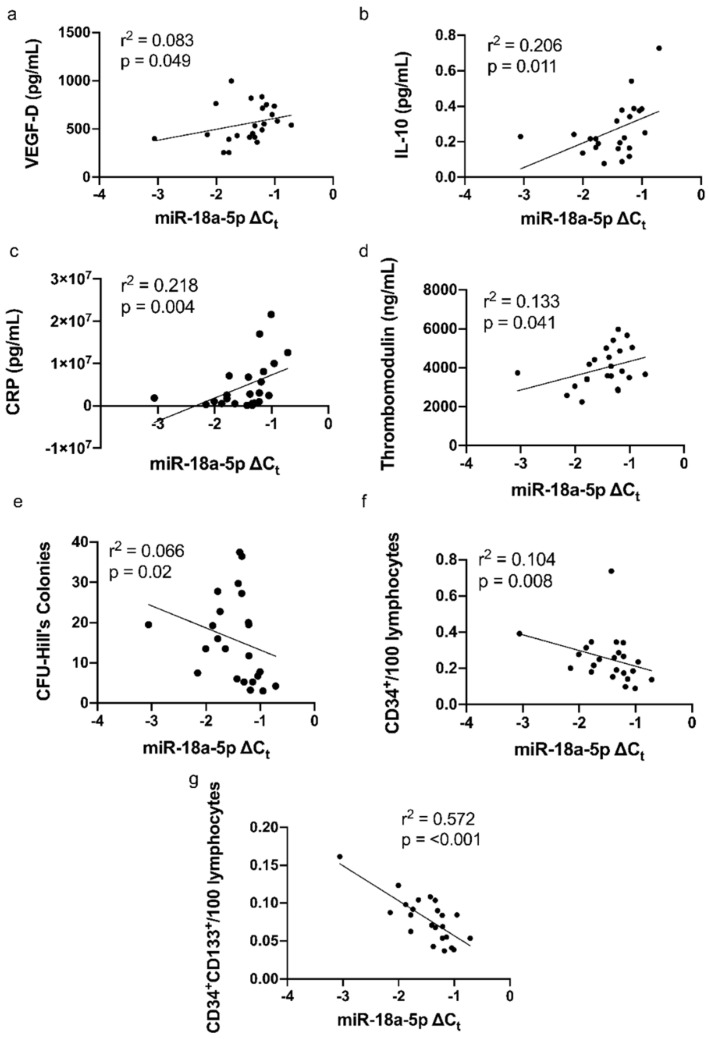
Correlation between miR-18a-5p expression in CFU-Hill’s colonies with the levels of (**a**) VEGF-D (r^2^ = 0.083, *p* = 0.049), (**b**) IL-10 (r^2^ = 0.206, *p* = 0.011), (**c**) CRP (r^2^ = 0.218, *p* = 0.004), (**d**) thrombomodulin (r^2^ = 0.133, *p* = 0.041) in the plasma, (**e**) CFU-Hill’s colonies per well (r^2^ = 0.066, *p* = 0.02), (**f**) CD34^+^/100 lymphocytes (r^2^ = 0.104, *p* = 0.008), and (**g**) CD34^+^CD133^+^/100 lymphocytes (r^2^ = 0.572, *p* < 0.001). Linear regression analysis was performed to assess the correlation. VEGF: vascular endothelial growth factor; IL: interleukin; CRP: C−reactive protein; CFU: colony-forming unit; CD: a cluster of differentiation.

Univariate linear regression analysis showed that miR-18a-5p was positively correlated with VEGF-D (r^2^ = 0.083, *p* = 0.049, Figure 4a), IL-10 (r^2^ = 0.206, *p* = 0.011, Figure 4b), CRP (r^2^ = 0.218, *p* = 0.004, Figure 4c) and thrombomodulin (r^2^ = 0.133, *p* = 0.041, Figure 4d), and negatively with CFU-Hill’s colonies (r^2^ = 0.066, *p* = 0.02, Figure 4e), CD34^+^/100 lymphocytes (r^2^ = 0.104, *p* = 0.008, Figure 4f) and CD34^+^CD133^+^/100 lymphocytes (r^2^ = 0.572, *p* < 0.001, Figure 4g).

In addition, the present study observed no statistically significant association between miR-18a-5p with VEGF-C, TNF-α, IFN-γ, PIGF, b-FGF, Tie-2, sFlt-1, sICAM-1, sICAM-3, E-selectin, P-selectin, IL-6, IL-7, IL-8, IP-10, and IL-16.

### 3.5. miR-18a-5p Molecular Targets and Functional Pathways

The impact of ingenuity pathway analysis (IPA), a knowledge-based database, was used to analyse and predict miR-18a-5p-mRNA targets to better understand the miRNA-mRNA interaction in the implication of CVD. By inputting hsa-miR-18a-5p, IL-10, CRP, VEGF-D, thrombomodulin, along with glucose to demonstrate a diabetic state into IPA software, miR-18a-5p was predicted to enhance the progression of atherosclerosis, infarction, and inflammatory response whilst inhibiting angiogenesis and blood coagulation. 

MiR-18a-5p was proven to inhibit the mRNA expression of insulin-like growth factor 1 (*IGF1*) [31]; it was also verified that the upregulation miR-18a-5p would inhibit mRNA expression of estrogen receptor 1 (*ESR1*), hypoxia-inducible factor-1 subunit alpha (*HIF-1α*), cellular communication network factor 2 (*CCN2*), and protein inhibitor of activated STAT 3 (*PIAS3*) [27,32,33,34]. 

**Figure 5:**
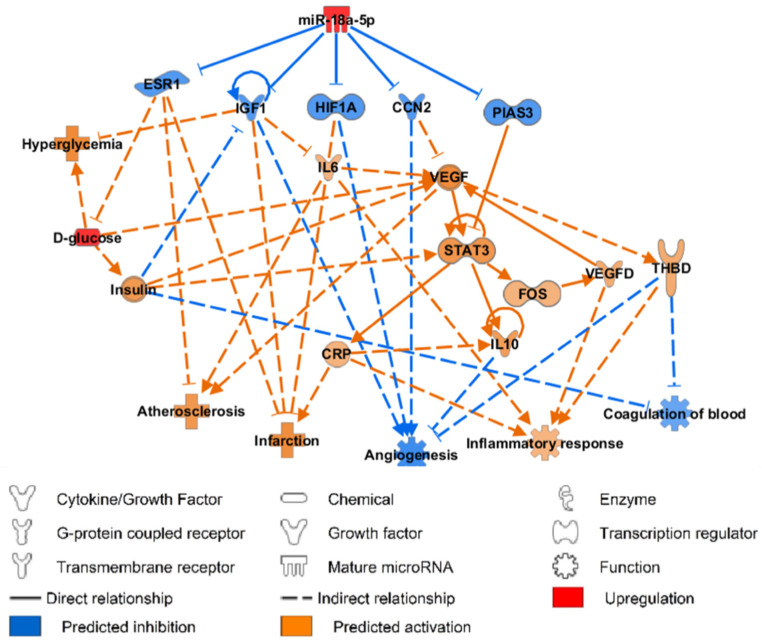
IPA prediction network of miR-18a-5p using our data and its mRNA targets and cytokines supporting its involvement in cardiovascular disease. *CCN2*: communication network factor 2; CRP: C−reactive protein; *ESR*: estrogen receptor 1; *FOS*: Fos proto-oncogene; *HIF-1α*: hypoxia-inducible factor-1 subunit alpha; *IGF1*: insulin-like growth factor 1; IL: interleukin; *PIAS3*: protein inhibitor of activated STAT 3; IL: interleukin; THBD: thrombomodulin; VEGF: vascular endothelial growth factor.

The inhibition of *CCN2* and *PIAS3* was predicted to activate the expression of signal transducer and activator of transcription 3 (*STAT3*), which then further augments the activation of CRP and IL-10. CRP was predicted to activate IL-10, therefore, inhibiting angiogenesis (Figure 5).

### 3.6. miR-18a-5p Molecular Targets and Functional Pathways Following Metformin Intervention

**Figure 6:**
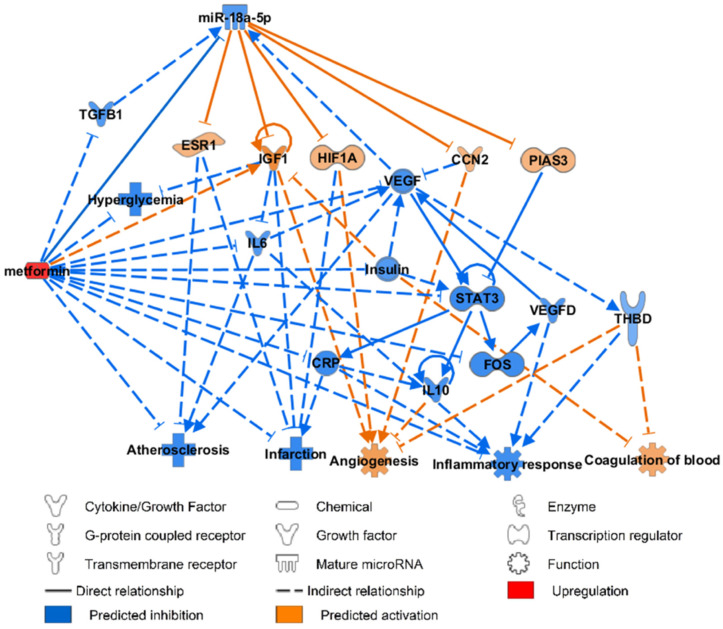
IPA prediction network of miR-18a-5p molecular targets and functional pathways following metformin intervention. *CCN2*: communication network factor 2; *CRP*: C−reactive protein; *ESR*: estrogen receptor 1; *FOS*: Fos proto-oncogene; *HIF-1α*: hypoxia inducible factor-1 subunit alpha; *IGF1*: insulin-like growth factor 1; *IL*: interleukin; *PI*AS3: protein inhibitor of activated STAT 3; *TGFβ1*: transforming growth factor beta 1; *THBD*: thrombomodulin; *VEGF*: vascular endothelial growth factor.

The effect of metformin on vascular indicators has been previously published by us [6]. Metformin inhibited miR-18a-5p expression, which would in turn upregulate the mRNA expression of *ESR1*, *IGF1*, *HIF-1α*, *CCN2*, and *PIAS3* (previously downregulated). The effect of metformin on miR-18a-5p was predicted to occur via *TGFβ1* and *VEGF* nodes [35,36,37,38]. Functional pathway analysis predicted that metformin therapy would inhibit the development of atherosclerosis, infarction, and attenuate inflammatory response; it was also predicted that metformin would aid angiogenesis and blood coagulation (Figure 6).

## 4. Discussion

This is the first study investigating miRNA expression in CFU-Hill’s colonies. Our study validated animal research on the anti-angiogenic properties of miR-18a-5p as we observed the upregulation of miR-18a-5p expression in CFU-Hill’s colonies, and its negative association with vascular health indices in patients with subclinical CVD. 

We confirmed that well-controlled T1DM had characteristics of subclinical CVD, as indicated by significantly increased of CRP and cytokines; moreover, we observed reduced CFU-Hill’s colony count, a lower number of cEPCs, and elevated thrombomodulin.

### 4.1. miR-18a-5p Expression in T1DM Patients

We have shown for the first time that miR-18a-5p in CFU-Hill’s colonies is upregulated in T1DM. MiRNA-18a-5p has been suggested to serve an important role in various biological activities leading to the pathogenesis of vascular diseases, as significantly upregulated miRNA-18a-5p was previously reported in the serum of patients with stent stenosis and in animal models with carotid artery injury [26]. 

The lack of differential expression of miR-18a-5p in our T1DM patients in plasma samples compared to healthy controls is in concordance with previous groups that showed no change in the expression of miR-18a-5p in plasma from patients with long-duration of T1DM and T2DM [39,40,41].

However, in another study miR-18a-5p was raised in sera from children with recent-onset T1DM and patients with early-onset diabetes suggesting that the onset of type 1 diabetes may coincide with raised serum miR 18a-5p expression [42,43]; this finding is in line with the prediction demonstrated in IPA analysis in our study showing indirect activation of hyperglycemia due to increased miR-18a-5p level. No comparison on miR-18a-5p expression in T1DM in other tissues is possible due to the lack of studies.

In this study, our ROC curve analysis demonstrated the potential utility of miR-18a-5p as a strong diagnostic biomarker for T1DM/subclinical CVD with an AUC of 0.889 (*p* = 0.002), 77.78% sensitivity and 100% specificity. We hypothesized that the difference of miR-18a-5p expression could be associated with the presence or absence of hyperglycemia; thus, we assessed the cut-off value of HbA1c to discriminate at which value the upregulation of miR-18a-5p occurred. The HbA1c value of 44.5 mmol/mol (6.2%) achieved from ROC analysis indicated an increased CVD risk within the pre-diabetes range (HbA1c 6.0–6.49%). Our findings emphasize the utilization of miR-18a-5p as a reliable non-invasive biomarker of increased CVD risk even in the absence of clinical CVD.

In contrast to our finding, another study reported downregulated serum miR-18a in T2DM, suggesting that miR-18a might improve insulin sensitivity via inhibition of PTEN, a known negative regulator of insulin sensitivity [44]. The discrepancies between studies may be partially explained by the difference in the population studied and the tissues/samples used to assess miR-18a-5p expression.

### 4.2. The Association between miR-18a-5p and CFU-Hill’s Colonies

In the present study, anti-angiogenic miR-18a-5p expression negatively correlated with CFU-Hill’s colonies, which have been demonstrated by experimental and clinical studies as an indicator of vascular health [8]. A reduced level of CFU-Hill’s colonies was significantly associated with an increased risk of developing the first major cardiovascular event and independently predicted coronary artery disease progression [28,45]. 

Given that we measured miR-18a-5p in CFU-Hill’s colonies one should expect a direct correlation between the two measurements; however, we found a negative correlation. Therefore, it could be postulated that the number of CFU-Hill’s colonies is reduced in an auto- and paracrine manner. Furthermore, this may suggest that miR-18a-5p is anti-angiogenic in T1DM since CFU-Hill’s (CD115) involved in EPC mobilization and angiogenesis in vivo [16]. 

### 4.3. The Association between miR-18a-5p and Vascular Health

T1DM has been demonstrated to be closely related to abnormal vascular findings, suggesting that preclinical CVD can be observed to a greater extent among T1DM patients [6,9,46,47]. Our study demonstrated an elevated level of thrombomodulin and a lower number of circulating CD34^+^ and CD34^+^CD133^+^ stem cells in T1DM patients. 

Our group is the first to report a positive linear relation between miR-18a-5p and thrombomodulin. The role of thrombomodulin in CVD is complex. Studies have demonstrated the anti-inflammatory effect of thrombomodulin, particularly the lectin-like domain of thrombomodulin [48]; however, elevated thrombomodulin may also suggest elevated soluble fragments of thrombomodulin from damaged endothelial cells [49]. In patients with existing atherosclerotic disease or children without soluble thrombomodulin was positively associated with future CHD events and arteriosclerosis progression, which may reflect the degree of associated inflammation and endothelial damage [50,51]. Furthermore, IPA prediction revealed upregulated miR-18a-5p activating thrombomodulin via VEGF activation; this finding is in line with a study demonstrating that VEGF upregulated thrombomodulin expression, thus enhancing endothelial cell adhesion and tube formation [52]; this suggests that elevated thrombomodulin in our patients serves as a compensatory mechanism.

We are the first to demonstrate a direct inverse relationship between miR-18a-5p and circulating CD34^+^ and CD34^+^CD133^+^ cells; these cells have been demonstrated to have the proliferative and angiogenic capacity, which may contribute to neo-angiogenesis [12,53]. As CFU-Hill’s colonies are involved in EPC mobilization, reduced CFU-Hill’s colonies in our patients might disrupt EPC mobilization, resulting in lower levels of CD34^+^ and CD34^+^CD133^+^ cells [16]. A study on CD34^+^ cell differentiation reported that miR-18a was significantly upregulated during erythropoiesis but not in granulopoiesis; this might indicate the role of miR-18a in bone marrow regulation [54]. 

Our findings are consistent with lower levels of circulating CD34^+^ and CD34^+^CD133^+^ cells reported in T1DM patients with history of microalbuminuria compared to T1DM patients without. Microalbuminuria was discerned as a marker of vascular injury and a risk factor for CVD; hence, circulating progenitor cells were observed to be lower in individuals with higher risk of CVD [55]. Similarly, in a cohort of 187 T2DM patients monitored over a 6-year period, baseline CD34^+^ and CD34^+^CD133^+^ cells were lower among those with incident cardiovascular events compared to those without [56]. In addition, a previous study reported that the duration of diabetes, and not the magnitude of hyperglycemia (HbA1c), contributed to CD34^+^ cell number [57]. 

### 4.4. The Association between miR-18a-5p and Inflammatory Markers

Our findings showed positive linear relation between miR-18a-5p and VEGF-D, IL-10, and CRP levels; this is consistent with IPA analysis predicting upregulated miR-18a-5p leading to VEGF-D activation via STAT3 and subsequent FOS activation. VEGF-D was predicted to further aggravate inflammatory response. VEGF-D is a member of the VEGF family, which plays an important role in lymphangiogenesis, endothelial cell growth, and angiogenesis [58]. Increased plasma VEGF-D has been associated with a higher incidence of atrial fibrillation, CVD and heart failure [59,60]; however, the positive association with heart failure was suggested to be a compensatory mechanism by expanding lymphatic capacity to eliminate excess fluid [60]. In diabetes, it has been suggested that downregulated VEGF receptor (VEGFR) and impaired downstream signal transduction are the contributing factors leading to reduced neoangiogenesis and compensatory elevated VEGF levels [61]. 

CRP has been well established to serve as a sensitive marker of acute inflammation, and recent studies have demonstrated its significance in chronic inflammatory diseases including CVD and diabetes mellitus [62]. Several lines of evidence suggest that CRP may directly contribute to the inflammatory process of atherosclerosis [63,64]. CRP has been demonstrated to participate in thrombus formation and significant expression of adhesion molecules in ECs, therefore contributing to the development of atherosclerosis [64,65]. 

We suggested that elevated IL-10 in T1DM could serve as a compensatory response to the inflammatory nature to counterbalance elevated CRP, IL-8, and TNF-α, as previously reported [11,47]. Due to its anti-inflammatory property, the administration of IL-10 might be a potential approach for the management of T1DM and CVD. To date, clinical data on therapies targeting IL-10 for CVD is currently unavailable but animal experiments have supported the prospect of IL-10 therapy. In rats with heart failure following MI, IL-10 therapy significantly improved post-MI left ventricular function [66]. The anti-inflammatory role of IL-10 was suggested to contribute to reduced levels of IL-6 and TNF-α. Furthermore, in ischemia-reperfusion injury animal models, IL-10 mitigated inflammation and cardiomyocyte death by attenuating oxidized phospholipids-mediated lipid metabolic responses [67]; these findings in corroboration with our study support the potential of IL-10 therapy for the treatment of inflammatory diseases and conditions including CVD. 

The positive association with IL-10 is confirmed by IPA analysis as overexpressed miR-18a-5p was predicted to upregulate IL-10 via IL-6, VEGF and STAT3 activation. A study showed that overexpressed miR-18a enhanced IL-6 response, which then led to increased miRNA cluster miR-17/92 in a positive feedback loop; this feedback loop leading to an amplified inflammatory process was facilitated by the repression of PIAS3, a repressor of STAT3 [68]; this finding is in concordant with our IPA pathway analysis. IL-6 has been shown to induce IL-10 and cause a delayed increase in CRP [69]; this may explain how upregulated miR-18a-5p led to elevated IL-10 and CRP in the present study. In another study, serum IL-10 was significantly elevated in patients with acute coronary syndrome compared to HCs [70]; however, since IL-10 is most widely known as an anti-inflammatory cytokine, this elevation may occur as a self-protective response to the pro-inflammatory nature of atherosclerosis to prevent excessive inflammation [71]. 

Similarly, a positive association with CRP confirms that miR-18a-5p is directly correlated to an inflammatory state. Here, we showed for the first time a direct relationship between miR-18a-5p and CRP, a protein synthesized by hepatocytes in response to inflammatory cytokines. CRP has been well established to serve as a sensitive marker of acute inflammation, and recent studies have also demonstrated its significance in chronic inflammatory diseases like CVD and diabetes mellitus [62]. We hypothesize that upregulation of miR-18a-5p would be pro-inflammatory, which was supported by IPA prediction demonstrating its activation of inflammatory response; however, it is unclear if the inflammatory state leads to increased miR-18a-5p expression.

Taken together, we postulate that the upregulation of miR-18a-5p is pro-inflammatory, which is concordant with IPA prediction demonstrating the activation of inflammatory response.

### 4.5. The Association between miR-18a-5p and HbA1c

MiR-18a-5p was negatively correlated with HbA1c, and this could suggest that diabetic control may play a role in the generation and regulation of miR-18a-5p. Our result concurs with a study investigating the expression of stress-related miRNAs in individuals with newly diagnosed T2DM demonstrating that circulating miR-18a correlated with impaired fasting glucose and insulin resistance [72]; moreover, downregulated plasma miR-18a-5p was reported in individuals with pre-diabetic glucose tolerance impairment compared to either T2DM diagnosed patients or those with normal glucose tolerance; this underlines that pre-diabetes, characterized by recurrent glucose variations, might present deleterious effects to target tissues and affect regulation of miRNA more drastically compared to chronic hyperglycemia in T2DM [39]. 

### 4.6. miR-18a-5p Expression Following Metformin Therapy

In this study we showed for the first the effect of metformin on miRNA expression in CFU-Hill’s colonies. Metformin has been recommended as the first-line drug for patients with type 2 diabetes mellitus (T2DM) due to its cardioprotective effect [73], and recently National Institute for Health and Care Excellence (NICE) recommended prescribing metformin as adjunctive therapy to insulin in overweight or obese patients with T1DM [74]. In the REMOVAL trial, T1DM patients receiving metformin displayed a significant reduction in mean change/year in maximal carotid-intima media thickness (cIMT, a surrogate measure of cardiovascular risk) in comparison to the placebo group [75]. We have previously published that metformin therapy has a potential cardio-protective effect in T1DM patients through improving CFU-Hill’s colonies, cEPCs, cECs, PACs count and cell adhesion [6]. 

Our results indicate that metformin therapy led to the downregulation of miR-18a-5p expression in CFU-Hill’s colonies, normalizing its expression to the level seen in HC. Similarly, long-term metformin exposure to senescence-associated miRNA and miRNA iso-form (iso-miR) in HUVECs was demonstrated to downregulate miR-18a-5p [76]. 

Our study strengthened the evidence to support metformin therapy as a cardioprotective drug. The inhibition of miR-18a-5p by metformin was proven to occur via TGFβ1 and VEGF [30,31,32,33]. Furthermore, IPA analysis explicated the mechanism on how metformin may reverse the cardio-detrimental effects of miR-18a-5p via activation of mRNA *IGF1*, *ESR1*, *HIF-1α*, *CCN2*, and *PIAS3* [27,31,32,33,34].

### 4.7. Prediction Model: Functional Pathway Analysis of miR-18a-5p in Relation to Cardiovascular Function 

IPA analysis summarized our findings, that the upregulation of miR-18a-5p would directly inhibit *IGF1*, *ESR1*, *HIF-1α*, *CCN2*, and *PIAS3*. The inhibition of these mRNAs was predicted to subsequently activate the expression of IL10, CRP, VEGF-D, and THBD; this prediction is in accordance with our miRNA correlation analysis, which showed that the upregulation of miR-18a-5p was positively correlated with the levels of IL10, CRP, VEGF-D, and THBD. 

In addition, the upregulation of miR-18a-5p was predicted to be cardio-detrimental via the activation of atherosclerosis, infarction, and inflammatory response, as well as the inhibition of angiogenesis and blood coagulation. Metformin was predicted by IPA to be cardio-protective by reversing the cardio-detrimental effects of miR-18a-5p upregulation. Inhibition of *TGFβ1* and *VEGF* was proven to result in the subsequent reduction of miR-18a-5p level. The cardioprotective action of metformin via *TGFβ1* inhibition is in line with the previous molecular dynamic simulations showing that metformin directly interacts with *TGFβ1*, therefore inhibiting its binding with receptors and attenuating its downstream signalling [77]; moreover, the IPA predicted inhibition of *VEGF* is also in line with a prior diabetic mice model study, which demonstrated that metformin attenuated VEGF signaling activation in the development of diabetic retinopathy [78]. Here, we showed for the first time that metformin was proven to result in indirect activation of IGF1, ESR1, HIF-1α, CCN2, and PIAS3 via miR-18a-5p downregulation.

As CFU-Hills derive from circulating peripheral blood mononuclear cells this strengthens the link between the effect of miRs expressed in CFU-Hills on circulating cytokines or growth factors in our study. Furthermore, the evidence that IPA modeling is not only generated by predictions but also is biologically relevant comes from animal models listed in this discussion.

Finally, we can speculate that the mechanism of how miR can influence vascular structures studied by us could also include regulation of *FGF1*, *HIF-1α*, syndecan4 or AKT/ERK signaling pathway in concerned tissue [25,26,27]. Further research is necessary to validate our findings and confirm the mechanisms.

### 4.8. Clinical Applications of Our Research for CVD 

Our research is concordant with the depth and breadth of ongoing research into CVD therapies. In addition to miRNA-based and cytokine-based therapies, we strengthened the evidence to support targeting downstream target genes implicated in CVD. 

#### 4.8.1. miRNA-Based Therapy

The inhibition of miR-18a-5p may present a novel therapy in managing T1DM and its cardiovascular complications. Despite emerging evidence on the role of miRNA in CVD, limited studies have been conducted on the role of miR-18a-5p in CVD development and progression. In an acute myocardial infarction (AMI) model, rat cardiomyocytes transfected with miR-18a inhibitor were demonstrated to upregulate brain-derived neurotrophic factor expression, thus ameliorating cardiac ischemic injury and offering protection against AMI [79]. 

#### 4.8.2. IL-10 

The elevation of IL-10 in T1DM could serve as a compensatory response to the inflammatory nature to counterbalance elevated CRP, IL-8, and TNF-α [11,47]. Due to its anti-inflammatory property, IL-10 administration could be a potential therapeutic approach for the management of T1DM and CVD. To date, clinical data on IL-10 therapy for CVD is currently unavailable but animal experiments have supported its prospect. In rats with heart failure, IL-10 therapy significantly improved post-MI left ventricular function by reducing IL-6 and TNF-α, whilst in ischemia-reperfusion injury, IL-10 therapy reduced inflammation and cardiomyocyte death [66,67].

#### 4.8.3. VEGF-D 

VEGF-D is part of the VEGF family of growth factors, which play a key role in angiogenesis by promoting new vessel formation during vascular development and following an injury [80]. VEGF-D mRNA, protein levels, normally expressed in the heart were significantly overexpressed in both early and late stages of MI, whilst VEGFR-3 was expressed in newly formed vessels in the infarcted myocardium, suggesting that VEGF-D is involved in angiogenesis in the infarcted heart [81]. 

Our study reported higher VEGF-D levels in T1DM/subclinical CVD. Thus, this elevation is speculated to serve as a compensatory mechanism to induce angiogenesis. In patients with refractory angina, endocardial adenoviral injection of the VEGF-D gene has been shown to be well-tolerated and increase myocardial perfusion in areas with impaired perfusion reserve [82]. Overall, our data and others support the promise of VEGF-D therapy in managing CVD.

#### 4.8.4. Thrombomodulin 

Elevated thrombomodulin among T1DM/subclinical CVD is postulated to reflect the degree of damaged endothelial cells. Prior studies have explored the anti-inflammatory nature of thrombomodulin, and in a mouse carotid ligation model, exogenous administration of thrombomodulin reduced atherosclerosis and the formation of neointima [83]. In addition, a soluble form of thrombomodulin tested in a clinical trial was shown to improve disseminated intravascular coagulation and sepsis [84].

#### 4.8.5. IGF1 

IGF-1 has anti-inflammatory and anti-atherogenic roles, and IGF-1 receptor knockout mice have been shown to exhibit increased atherosclerotic burden, less stable plaque composition, and enhanced pro-inflammatory responses in macrophages [85]. Furthermore, IGF-1 has been demonstrated to stimulate the angiogenesis process and angiogenesis-related growth factors expression via PI3-kinase/Akt signaling pathway [86]. 

#### 4.8.6. ESR1 

An animal model study demonstrated that ESR1 knockout mice exhibited insulin resistance, glucose tolerance impairment and obesity [87]; moreover, Zhai et al. demonstrated the cardioprotective role of estrogen receptor-α, an estrogen receptor encoded by ESR1, by investigating ischemia-reperfusion injury in male estrogen receptor-α knockout mice; they reported more severe myocardial damage and higher incidence of ventricular arrythmias in knockout mice hearts compared to control hearts [88]. In addition, ESR1 has been reported to enhance the expression of VEGF-A mRNA in a HIF-1α-dependent manner, which results in enhanced angiogenesis and attenuated inflammation [89]. 

#### 4.8.7. HIF-1α

The overexpression of HIF-1α in mesenchymal stem cell-derived exosomes on hypoxia-pre-treated HUVECs demonstrated that the angiogenic function, migratory capacity, and proliferation of the hypoxia-injured cells were reversed by HIF-1α-overexpressed exosomes; moreover, they reported enhanced neovessel formation in the infarcted area of myocardial infarction model, demonstrating the cardio-protective role mediated by HIF-1α [90]. Furthermore, Li et al. demonstrated that the injection of mutant HIF-1α to the skeletal muscle of ischemic rabbits resulted in improved tissue perfusion, mature angiogenesis, and more histologically identifiable capillaries [91]. 

#### 4.8.8. CCN2

Increased myocardial expression of CCN2 has been demonstrated to confer cardio-protection by protecting the heart from ischemia-reperfusion injury via GSK-3ß pathway inhibition, phospho-SMAD2 activation, and gene expression reprogramming [92]; moreover, in a model of acute cardiomyopathy, transgenic mice with cardiomyocyte-specific CTGF overexpression displayed preserved cardiac function compared to wild-type control rats that exhibited significantly reduced systolic function [93].

#### 4.8.9. PIAS3

Overexpression of PIAS3 has been shown to attenuate oxidized low-density lipoprotein(ox-LDL)-induced inflammation and lipid accumulation, making PIAS3 a vital repressor of atherosclerosis [94]; moreover, in a rabbit atherosclerosis model, inhibition of STAT3 signaling pathway led to less upregulation of pro-inflammatory cytokines and reduced formation of atheromatous plaques [95]. 

#### 4.8.10. TGFβ1

In animal studies, mice subjected to transverse aortic arch constriction exhibited elevated level of TGFβ1 [96]; moreover, TGFβ1-deficient mice subjected to angiotensin II were demonstrated to attenuate angiotensin II-induced cardiac hypertrophy compared to wild-type mice that exhibited impaired cardiac function and over 20% increase in left ventricular mass [97]. In an experimental model of chronic Chagas’ heart disease, TGFβ-inhibitor therapy resulted in reduced fibrosis of cardiac tissue and improved cardiac recovery [98].

### 4.9. Contribution/Causation

Based on our correlation analysis, we identified possible contribution or causality generated from miR-18a-5p target genes binding site (Table 2). By utilizing TargetScan Human, release 7.1 and Diana-TarBase v8 databases, we explored probable target genes of interest for miR-18a-5p; moreover, the canonical pathway tool in IPA was used to establish the most relevant canonical pathways associated with the target genes. In addition, the top molecular targets and functional pathways for miR-18a-5p using miRDB and mirPath database, respectively, were summarized in the supplemental material (Tables S1 and S2).

MiR-18a-5p has an effect on multiple pathways (IPA), of which many are interrelated. MiR-18a-5p has binding sites in 5 target genes of interest. We observed that the highest number of binding sites was present among the HIF-1α pathway; it became apparent that miR-18a-5p has a causal role via the HIF-1α pathway, in which molecules including HIF-1α, IGF1, IL-6, STAT3, VEGF, and VEGF-D were found to participate in this pathway; this pathway was predicted to be involved in the development of angiogenesis and infarction. The next causal effect was via IGF1, which is involved in inflammation and hyperglycemia. We also observed the causal effect of miR-18a-5p via ESR1, which leads to atherosclerosis and infarction. Another causal role observed was via CCN2, which is involved in angiogenesis. Lastly, miR-18a-5p appears to have a causal effect on PIAS3, which was involved in infarction and inflammation.

**Figure 7:**
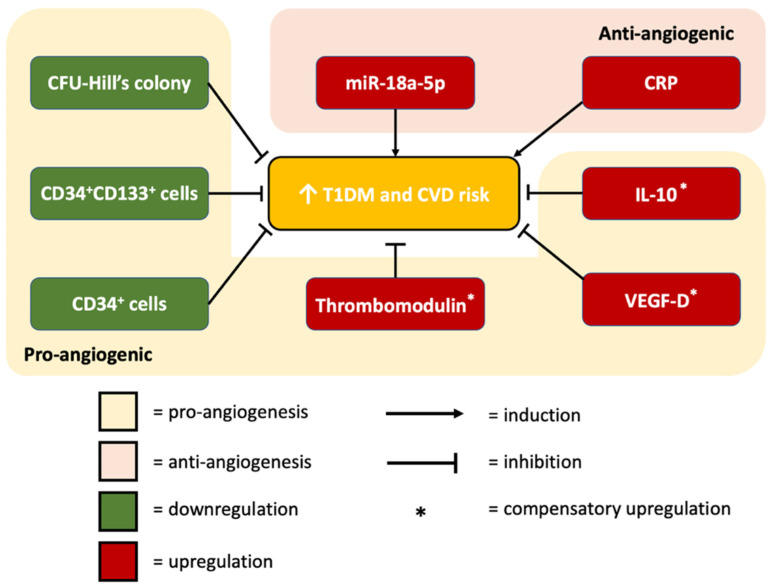
Summary of our study results in patients with subclinical CVD. The upregulated miR-18a-5p and CRP contributed to increased cardiovascular risks in T1DM patients, whereas reduced levels of CFU-Hill’s colony, CD34^+^ cells, and CD34^+^CD133^+^ cells were observed, indicating poor vascular health and repair. Elevated level of thrombomodulin reflected the degree of inflammation and endothelial damage leading to increased CVD risk. Elevated IL-10 and VEGF-D served as compensatory mechanism to counteract inflammatory conditions.

The summary of the present research is represented in Figure 7.

Given we have identified the potential therapeutic targets of metformin therapy, further studies are essential to prove the causal role of miR-18a-5p on the genes predicted by IPA.

### 4.10. Limitations

The limitation of our study is the technical constraints in attaining a sufficient amount of RNA from CFU-Hill’s colonies to analyze in parallel miRNA and mRNA in individual subjects and avoid pooling RNA samples for patients and HCs. Although the other limitation is the relatively small number of subjects studied, this is the first miRNA study ever carried out in CFU-Hill’s and should be treated as a pilot/feasibility study providing fundamental blocks for future research.

## 5. Conclusions

Our research-validated animal research on anti-angiogenic properties of miR-18a-5p as we observed upregulated miR-18a-5p among T1DM patients, whilst miR-18a-5p positively correlated with inflammatory markers and inversely with vascular health indices. Our findings highlight the promise of exploiting miR-18a-5p as an early, sensitive, non-invasive biomarker in diagnosing and monitoring CVD in clinical practice. As miR-18a-5p was predicted to inhibit genes IGF1, ESR1, HIF-1α, CCN2, and PIAS3 and act on cytokines, those targets could potentially be targeted for future CVD research in patients; moreover, the predicted cardioprotective mode of action of metformin provides consistent results with the activation of miR-18a-5p downstream target genes in animal CVD research; this, however, requires validation in clinical studies.

## Figures and Tables

**Table 1:** Antibodies used for enumeration of cEPCs and progenitor cells.

Antibody	Fluorochrome	Volume
CD34	PerCP-Cy5.5	20 μL
CD133	APC	5 μL
VEGFR2 (KDR)	PE	5 μL
CD144	FITC	10 μL
CD45	V500	5 μL

**Table 2:** The predicted consequential pairing of the target region in the transcript and miR-18a-5p.

Target Gene	Representative Transcript	Gene Name	Transcript Position	Predicted Consequential Pairing of Target Region Transcript (Top) and miRNA (Bottom)	Site Type
IGF1	ENST00000337514.6	Insulin-like growth factor 1	181–187 3′ UTR	(transcript) 5′ CUUUAGGAGUGAUUU**GCACCUU**G (miRNA) 3′ GAUAGACGUGAUCUA**CGUGGAA**U	7mer-8
ESR1	ENST00000440973.1	Estrogen Receptor 1	1938–1945 3′ UTR	(transcript) 5′ UAGUUUGUUUAAGAA**GCACCUUA** (miRNA) 3′ GAUAGACGUGAUCUA**CGUGGAA**U	8mer
HIF-1α	ENST00000323441.6	Hypoxia Inducible Factor 1 Subunit Alpha	409–415 3′ UTR	(transcript) 5′ AUCAUUUUAAAAAAU**GCACCUU**U (miRNA) 3′ GAUAGACGUGAUCUA**CGUGGAA**U	7mer-m8
CCN2 (CTGF)	ENST00000367976.3	Cellular Communication Network Factor 2 (Connective Tissue Growth Factor; CTGF)	1046–1052 3′ UTR	(transcript) 5′ AAAAGUUACAUGUUU**GCACCUU**U (miRNA) 3′ GAUAGACGUGAUCUA**CGUGGAA**U	7mer-m8
PIAS3	ENST00000393045.2	Protein Inhibitor of Activated STAT 3	774–780 3′ UTR	(transcript) 5′ GGCCUGGCUCAUUCU**GCACCUU**G (miRNA) 3′ GAUAGACGUGAUCUA**CGUGGAA**U	7mer-m8

TargetScan Human, release 7.1 (www.targetscan.org, accessed on 4 April 2022), Diana-TarBase v8, (http://carolina.imis.athena-innovation.gr/diana_tools/web/index.php?r=tarbasev8%2Findex, accessed on 4 April 2022) databases were used to predict the interaction sites between the transcripts and miR-18a-5p. Nucleotides in bold are predicted consequential pairing of target region transcript and miRNA.

## Supplementary Materials

The following supporting information can be downloaded at: https://www.mdpi.com/article/10.3390/biomedicines10092136/s1, Table S1: Top 10 molecular targets for miR-18a-5p; Table S2: Top 10 functional pathways for miR-18a-5p. References [99,100,101] are cited in the supplementary materials.
