# Supplementary material for: Upregulated miR-18a-5p in Colony Forming Unit-Hill’s in Subclinical Cardiovascular Disease and Metformin Therapy; MERIT Study
Source: Biomedicines. 2022 Aug 31;10(9):2136. doi: 10.3390/biomedicines10092136 (PMC9496122; doi:10.3390/biomedicines10092136)
Supplement: Supplementary file 1 [file biomedicines-10-02136-s001.zip › biomedicines-1858095-supplementary.pdf]

biomedicines

MDPI

Supplementary Material

Table S1. Top 10 molecular targets for miR-18a-5p.

Target Rank	Target Score	miRNA Name	Gene Symbol	Gene Description
1	99	hsa-miR-18a-5p	GLRB	glycine receptor beta
2	99	hsa-miR-18a-5p	PHC3	polyhomeotic homolog 3
3	99	hsa-miR-18a-5p	BBX	BBX, HMG-box containing
4	99	hsa-miR-18a-5p	RORA	RAR related orphan receptor A
5	98	hsa-miR-18a-5p	CDK19	cyclin dependent kinase 19
6	98	hsa-miR-18a-5p	NEDD9	neural precursor cell expressed, developmentally down-regulated 9
7	97	hsa-miR-18a-5p	DICER1	dicer 1, ribonuclease III
8	97	hsa-miR-18a-5p	PATJ	PATJ, crumbs cell polarity complex component
9	96	hsa-miR-18a-5p	FAM3C	family with sequence similarity 3 member C
10	96	hsa-miR-18a-5p	HIF-1α	hypoxia inducible factor 1 subunit alpha

miRDB - MicroRNA Target Prediction Database (http://mirdb.org/cgi-bin/search.cgi) [1, 2] .

Table S2. Top 10 functional pathways for miR-18a-5p.

Rank	KEGG Pathway	p-value	Number of genes
1	TGF-beta signaling pathway (hsa04350)	0.0014	2
2	Endocytosis (hsa04144)	0.0040	3
3	Colorectal cancer (hsa05210)	0.0071	2
4	Synthesis and degradation of ketone bodies (hsa00072)	0.0298	1
5	p53 signaling pathway (hsa04115)	0.0298	2
6	mTOR signaling pathway (hsa04150)	0.0298	2
7	Transcriptional misregulation in cancer (hsa05202)	0.0298	3
8	Chronic myeloid leukemia (hsa05220)	0.0362	2
9	Pancreatic cancer (hsa05212)	0.0415	2
10	Pathways in cancer (hsa05200)	0.0439	4

DIANA miRPath v.2.0: investigating the combinatorial effect of microRNAs in pathways [3].

References

1. Chen, Y.; Wang, X., miRDB: an online database for prediction of functional microRNA targets. *Nucleic Acids Res* **2020,** 48, (D1), D127-D131.
2. Liu, W.; Wang, X., Prediction of functional microRNA targets by integrative modeling of microRNA binding and target expression data. *Genome Biol* **2019,** 20, (1), 18.
3. Vlachos, I. S.; Kostoulas, N.; Vergoulis, T.; Georgakilas, G.; Reczko, M.; Maragkakis, M.; Paraskevopoulou, M. D.; Prionidis, K.; Dalamagas, T.; Hatzigeorgiou, A. G., DIANA miRPath v.2.0: investigating the combinatorial effect of microRNAs in pathways. *Nucleic Acids Res* **2012,** 40, (Web Server issue), W498-504.
